# Health equity and worker justice: the food supply chain during the COVID-19 pandemic: Linda Forst

**DOI:** 10.1093/eurpub/ckac129.407

**Published:** 2022-10-25

**Authors:** L Forst

**Affiliations:** School of Public Health, University of Illinois Chicago, Chicago, USA

## Abstract

The COVID-19 pandemic has brought into stark relief the health, safety and security threats facing people in the US and around the globe. As stay-at-home orders were instituted, most economic activities closed or required work from home; however, companies in the Food Supply Chain (FSC) continued to operate, considered “essential businesses.” The meatpacking and agriculture sectors in Illinois - 79,000 and 55,000 workers, respectively - are largely populated by (im)migrant workers, some on short-term work visas and many of whom are part of the informal (cash) economy. Only a tiny percentage of meatpacking and no agriculture businesses are unionized in Illinois or most of the USA. Initially, FSC workplaces were not re-configured for social distancing; sanitation and entry procedures were not instituted; workers were not offered personal protective equipment (PPE); and no plans were made for quarantine of infected workers or isolation of COVID-exposed workers. Lack of paid sick leave for these workers, who earn subsistence wages and whose employment is extremely precarious due to immigration status, added pressure for them to continue to work while infectious. Investigation and enforcement of health and safety was taken on by local health departments rather than the occupational safety and health enforcement agency. Finally, these workers, who live in congregate housing and share transportation, carried the virus from work to community and community to work. We review US laws related to immigration, labor, and occupational health and safety before and during the pandemic, as well as epidemiological data. We will highlight the changes required to address “work” in the web of contagion during an infectious disease epidemic. Protection of workers’ rights, as well as a clearer understanding of the integral connection between workplace and community, will be a likely “side effect” of these efforts.

